# Granulocyte-like myeloid derived suppressor cells (G-MDSC) are increased in multiple myeloma and are driven by dysfunctional mesenchymal stem cells (MSC)

**DOI:** 10.18632/oncotarget.7969

**Published:** 2016-03-07

**Authors:** Cesarina Giallongo, Daniele Tibullo, Nunziatina L. Parrinello, Piera La Cava, Michelino Di Rosa, Vincenzo Bramanti, Cosimo Di Raimondo, Concetta Conticello, Annalisa Chiarenza, Giuseppe A. Palumbo, Roberto Avola, Alessandra Romano, Francesco Di Raimondo

**Affiliations:** ^1^ Division of Hematology, A.O.U. Policlinico-OVE, Catania, University of Catania, Italy; ^2^ Department of Biomedical and Biotechnological Sciences, University of Catania, Catania, Italy; ^3^ Department of Biological, Geological and Environmental Sciences, University of Catania, Catania, Italy

**Keywords:** MM microenvironemnt, mesenchymal stem cells, G-MDSC, immune-suppression

## Abstract

Granulocytic-Myeloid-derived suppressor cells (G-MDSC) are increased in Multiple Myeloma (MM) patients but the mechanisms of G-MDSC generation are still unknown. There are many evidences of the role of mesenchymal stem cells (MSC) in promoting MM cell growth, survival and drug-resistance. We here used a specific experimental model *in vitro* to evaluate the ability of MSC to induce G-MDSC. We found that although MSC derived from healthy donors (HD), MGUS and MM were able to generate the same amount of MDSC, only MM-MSC-educated G-MDSC exhibited suppressive ability. In addition, in comparison with MSC derived from HD, MM-MSC produce higher amount of immune-modulatory factors that could be involved in MDSC induction. Compared to G-MDSC obtained from co-culture models with MSC from healthy subjects, both MGUS and MM-MSC-educated G-MDSC showed increase of immune-modulatory factors. However, only MM-MSC educated G-MDSC 1) up-regulated immune-suppressive factors as ARG1 and TNFα, 2) expressed higher levels of PROK2, important in angiogenesis and inflammatory process, and 3) showed ability to digest bone matrix.

Our data demonstrate that MM-MSC are functionally different from healthy subjects and MGUS-MSC, supporting an evolving concept regarding the contribution of MM-MSC to tumor development and progression.

## INTRODUCTION

Monoclonal gammopathies encompasse a spectrum of clinical variants ranging from monoclonal gammopathy of uncertain significance (MGUS) through smoldering Multiple Myeloma (MM), up to the most aggressive, symptomatic MM and plasma cell leukemia [1, 2]. There are several evidences indicating that development of MM is due not only to uncontrolled proliferation of plasma cells (PC) but also to change in the bone marrow (BM) microenvironment [3]. Here PC are hosted in special niches and receive multiple signals that maintain their long survival and exert a protective effect on drug-induced apoptosis, due to the secretion of soluble factors, such as IL6 and extracellular vescicles [4–6]. Within the microenvironment, the host immune system has a pivotal role for the PC growth, proliferation, survival, migration and resistance to drugs and is responsible for some clinical manifestations of MM [4, 7, 8].

Myeloid-derived suppressor cells (MDSC) include myeloid cells at different stage of maturation characterized by the ability to suppress immune responses, including T cell proliferation and cytokine production [9–11]. Several groups highlighted the emerging role of MDSC in MM pathogenesis and clinical behavior, and have documented their increase in both peripheral blood (PB) and BM of MM patients [4, 12–18].

Based on the expression of surface antigens and studies available in mice, two main subpopulations of MDSC can be distinguished: CD11b^+^ CD14^−^CD15^+^CD33^+^CD66b^+^HLA-DR^−^ granulocyte-like (G-MDSC) and CD14^+^CD15^−^HLA-DR^−^ monocyte-like (Mo-MDSC) [5, 19–21]. MDSC are able to inhibit the immune system by multiple mechanism, mostly through production of arginase 1 (ARG1), nitric oxidase synthase 2 (NOS2), reactive species of oxygen (ROS), cyclooxygenase 2 (COX2), transforming growth factor β (TGF-β) and immunosuppressive cytokines, such as IL6, IL10 and IL1β [22]. In addition, MDSC can induce regulatory T-cells [22] and differentiate in functional osteoclasts contributing to the formation of osteolytic lesions in solid tumors [23–25].

BM mesenchymal stem cells (MSC) are essential components in the formation and function of the BM microenvironment [26–28]. They are a heterogeneous population of stromal adult stem cells with an important role into the tumor microenvironment due to their immunosuppressive ability, such as the capacity to inhibit T cell activation and proliferation. Indeed, MSC can interfere with the recognition of tumor cells by immune system producing and releasing immunoregulatory factors as TGFβ, prostaglandin E2 (PGE2), tumor necrosis factor α (TNFα), indolamine 2, 3-dioxygenase (IDO), hemeoxygenase (HO), NOS2, ARG_1–2_, IL10 [29–32]. MSC express programmed death ligand 1 (PD-L1) that after its engagement with PD-1 expressed on T lymphocytes lead to the inhibition of T cell activation and proliferation with an inefficient immune response [29, 33].

Even though we and others have demonstrated increased levels of MDSC in PB from MM patients and characterized their immunosuppressive role [4, 12–16], the role of MSC in MDSC expansion and activation into the BM microenvironment remains unexplored.

In the present study, we investigated the role of MSC obtained from MGUS and MM patients on expansion and activation of G-MDSC compared to MSC from healthy donors (HD).

## RESULTS

### Increased frequency of G-MDSC in MM patients

**Figure 1:**
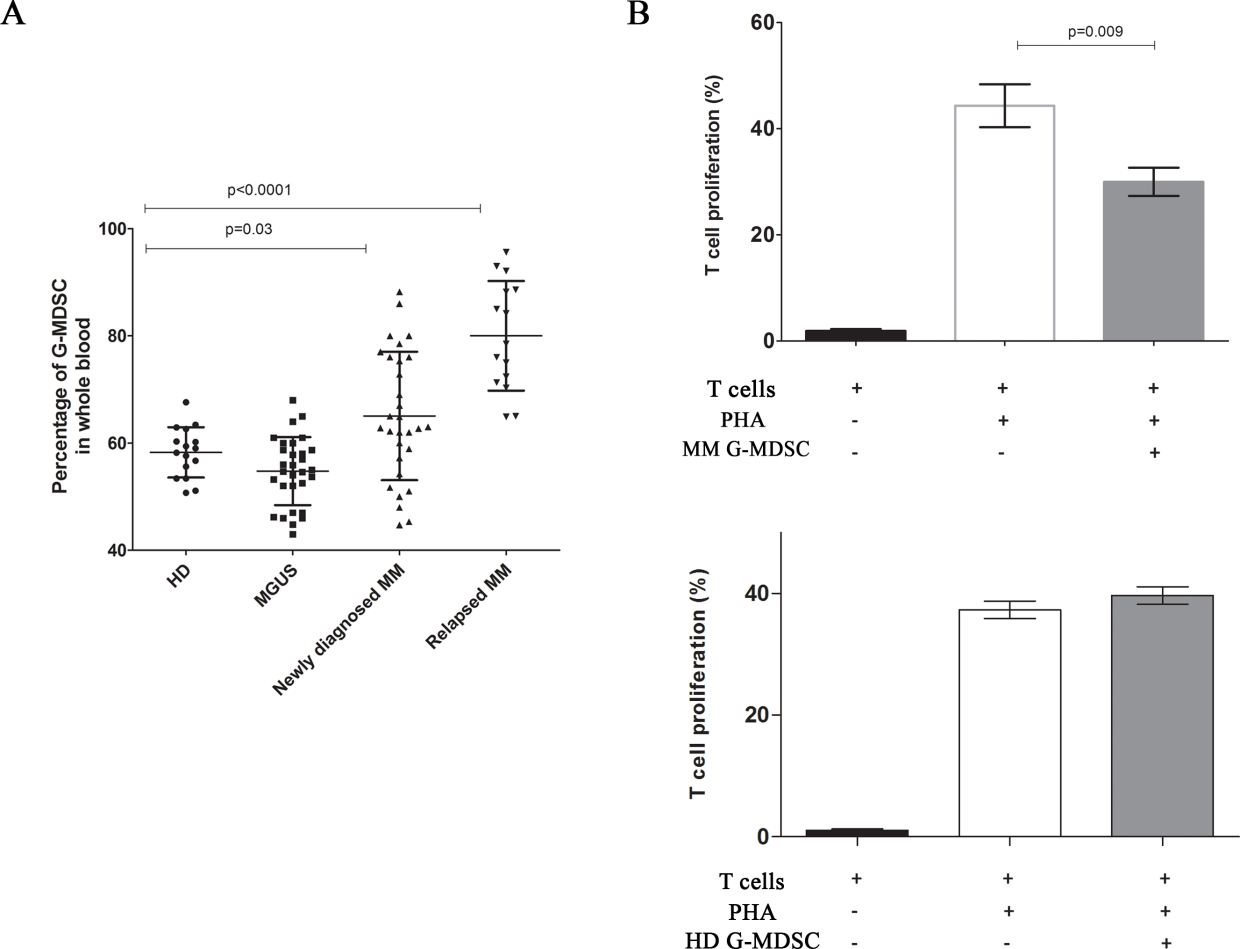
Increased frequency of G-MDSC in MM patients The percentage of circulating G-MDSC was quantified in the peripheral blood of healthy donors, MGUS and newly diagnosed or relapsed MM patients by flow cytometry (panel **A**). MM G-MDSC-mediated T-cells suppression was assessed in autologous co-cultures. Mean frequency of CD3+ CFSE dim cells ± SD from four independent experiments in duplicate is shown (panel **B**).

The frequency of G-MDSC (CD11b^+^CD15^+^CD14^−^HLADR^−^ cells) was evaluated in the PB of HD, MGUS and MM patients at diagnosis and relapsed using flow cytometry. Percentage of G-MDSC was significantly higher in PB of patients with newly diagnosed (65.1 ± 11.9%), and relapsed (80.1 ± 10.2%) MM compared to MGUS (54.7 ± 6.3) and healthy subjects (58.2 ± 4.6%) (*p* = 0.03 and *p* < 0.001 respectively) (Figure 1A).

We next evaluated the immune-suppressive activity of MDSC. For this purpose, we incubated G-MDSC obtained from MM and HD with autologous CFSE-labeled T cells and we found that only MM G-MDSC were able to reduce autologous T cells proliferation (44.3 ± 2.3% vs 30.0 ± 1.5%, *p* = 0.009) (Figure 1B).

### MM-MSC promote induction of MDSC in the microenvironment of MM patients

We next assessed the role of tumor microenvironment on expansion and activation of MDSC, focusing our attention on MSC.

**Figure 2:**
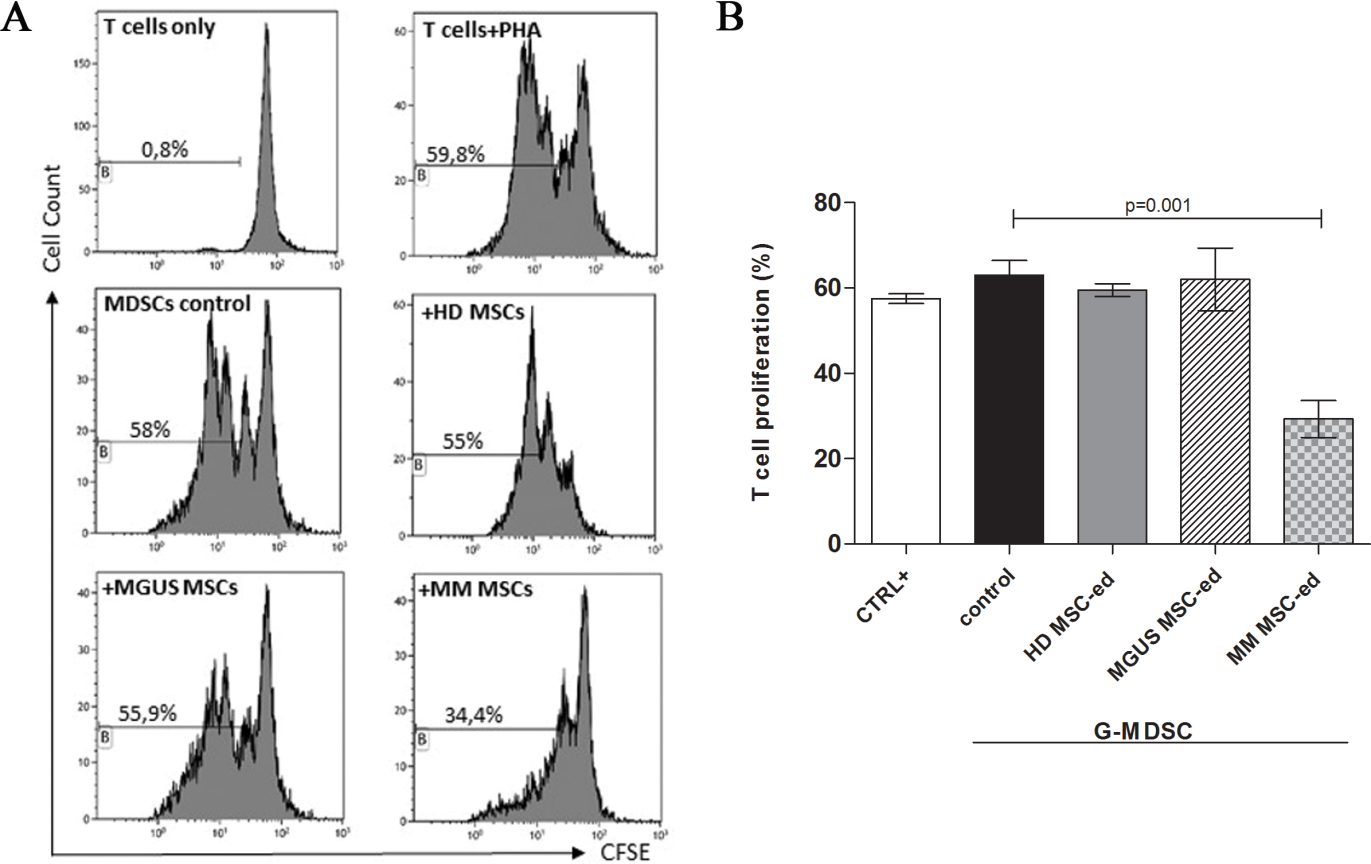
MM MSC educated G-MDSC are immunosuppressive MSCed-G-MDSC were analyzed for their immune-suppressive activity against autologous T-cells. Representative flow cytometry dot-plots show the gating strategy for each experimental condition (panel (**A**). Only MM MSCed-G-MDSC exhibited suppressive effects compared to G-MDSC control (*p* < 0.001, panel (**B**). The data represent mean ± SD of all analyzed co-cultures in triplicate.

To explore their influence in the induction of MDSC, PBMC from healthy donors were co-cultured with HD-, MGUS- or MM-MSC for one week. Then, the frequency of G-MDSC in PBMC was analyzed before cell magnetic separation. Both HD-, MGUS- and MM-MSC accumulated similar small amount of G-MDSC (Supplementary Figure 1). Next, we analyzed immune-suppressive activity of MSC-educated G-MDSC (MSCed-G-MDSC). These cells were obtained with magnetic cell separation and the G-MDSC phenotype was confirmed by cytofluorimetric analysis (Supplementary Figure 2). Interestingly, we found that only MM-MSCed-G-MDSC were able to suppress T-cells proliferation (*p* = 0.001), while this suppressive activity was not recorded for MGUS-MSCed-G-MDSC or HD-MSCed-G-MDSC or G-MDSC control (isolated from PBMC cultured in medium alone) (Figure 2).

Therefore, even if HD-, MGUS- and MM-MSC are able to generate similar amount of G-MDSC cells, only MM-MSCed-G-MDCS exhibited suppressive effect on T cell proliferation.

### Molecular regulators of MM MSC-mediated MDSC activation

**Figure 3:**
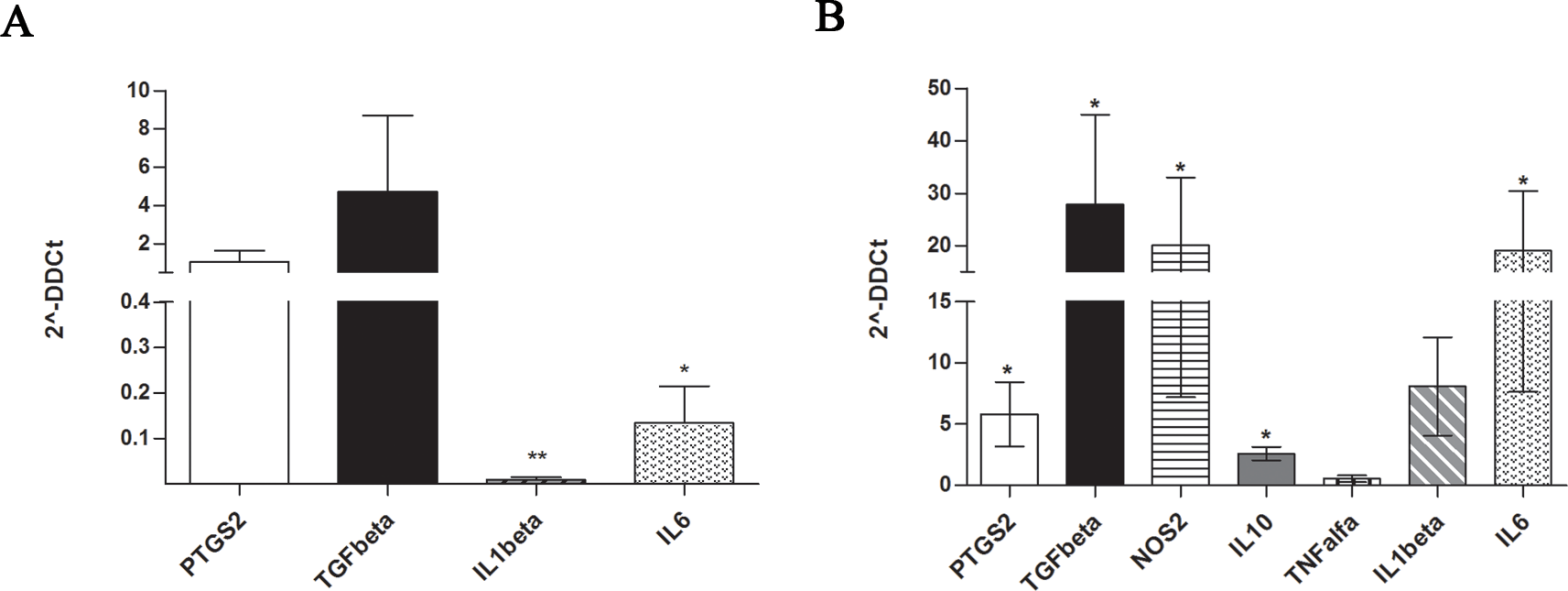
Expression of immune-modulatory factors by MM-MSC at Time 0 (A) and after 48 h of co-culture with PBMC (B) Only after incubation with PBMC, MM-MSCs showed statistically significant up-regulation of PTGS2, TGFβ, NOS2, IL10 and IL6 expression (*p* < 0.05) compared with HD-MSCs (calculated value of 2^-ΔΔCt in HD-MSC was 1).

In many cancers, it has been demonstrated that tumor-associated microenvironment produces a large amount of immune-modulating factors involved in reprogramming immature myeloid cells to become immunosuppressive G-MDSC and to attract them at the tumor sites [36]. These immunomodulatory factors include PTGS2, TGFβ, NOS2, IL10, TNFα, IL1β, and IL6. Therefore, we first analyzed their expression by MM-MSC compared to HD-MSC at Time 0. A great variability of expression was observed among the patients, but no up-regulation of the genes above described was observed (Figure 3A). After 48 h of co-culture with PBMC, MM-MSC showed statistically significant up-regulation of PTGS2 (5.8 ± 5, *p* = 0.018), TGFβ (27.8 ± 34, *p* = 0.03), NOS2 (20 ± 25.8, *p* = 0.04), IL10 (19 ± 1, *p* = 0.017) and IL6 (40.7 ± 22, *p* = 0.02) expression compared with HD-MSC (Figure 3B), suggesting that MM-MSC are functionally different from HD-MSC and are able to produce higher amount of immunomodulatory factors that could be involved in MDSC generation.

### Gene expression changes in MM MSC-educated G-MDSC

**Figure 4:**
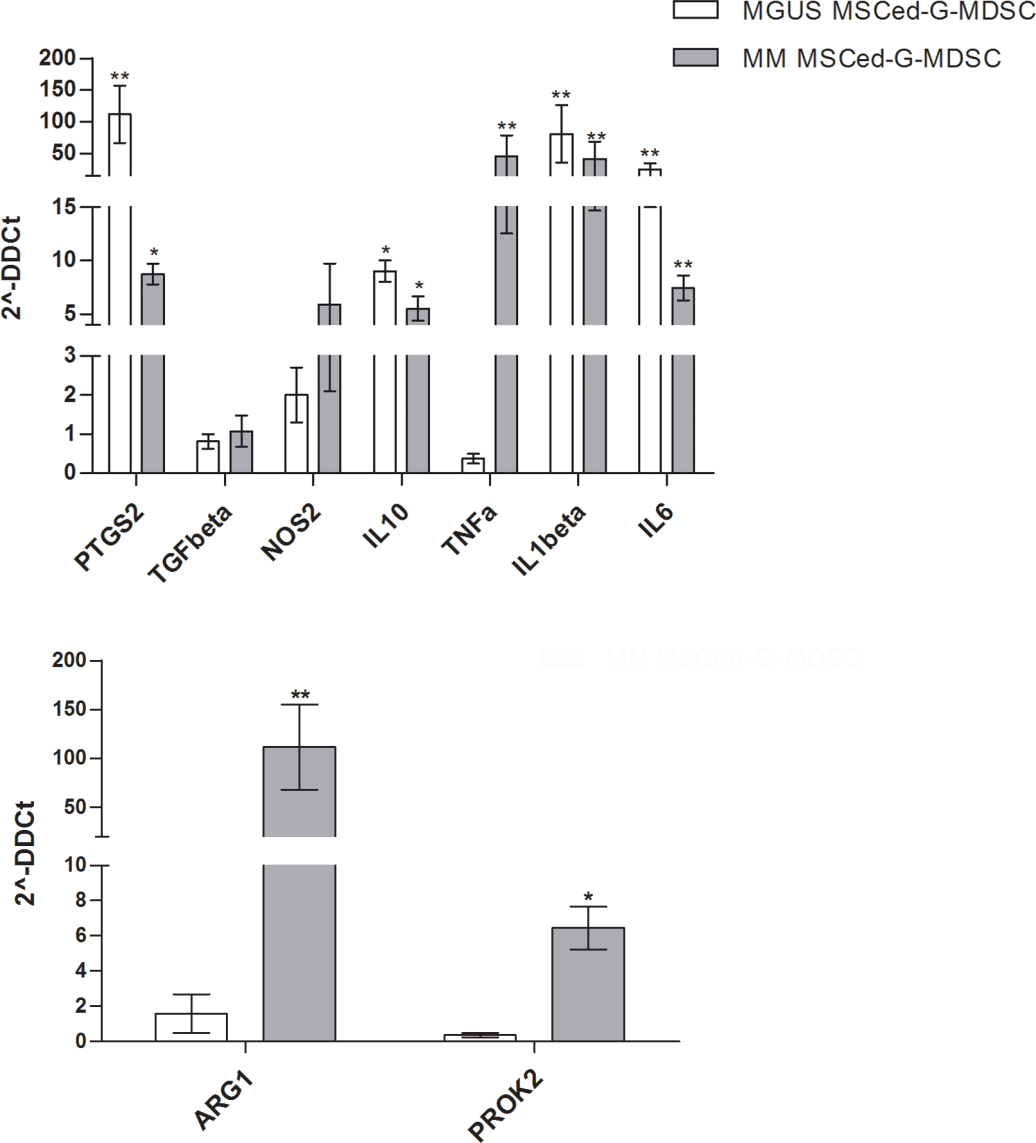
Expression of immune-modulatory and pro-angiogenic factors by MSCed-G-MDSC The graphs report fold change values in gene expression of indicated genes (normalized to HD MSCed-G-MDSC) in MM- and MGUS-MSCed-G-MDSC. Only MM-MSCed-G-MDSC significantly upregulated TNFα, ARG1 and PROK2. Calculated value of 2^-ΔΔCt in HD MSCed-G-MDSC was 1.

Before incubation with T cells, the relative expression levels of our set of immune modulatory factors was also investigated in MM- and MGUS-MSCed-G-MDSC compared to HD-MSCed-G-MDSC. With the exception of TGFβ, all the other immune-modulatory factors were found up-regulated in both MGUS- and MM-MSCed-G-MDSC, although up-regulation of TNFα (45.7 ± 28.8, *p* = 0.002) was almost exclusive of MM MSCed-G-MDSC (Figure 4).

We also found that only MM-MSCed-G-MDSC significantly upregulated PROK2 (6.4 ± 1.7, *p* = 0.03) and ARG1 (111.5 ± 50.2, *p* = 0.001). PROK2 is a molecule involved in angiogenesis and inflammatory process [37, 38], while ARG1 is an enzyme with a key role in immunosuppression. Releasing ARG1, myeloid cells deplete L-arginine and profoundly suppress T cell immune response [39].

### MM-MSC-educated G-MDSC can digest bone matrix

**Figure 5:**
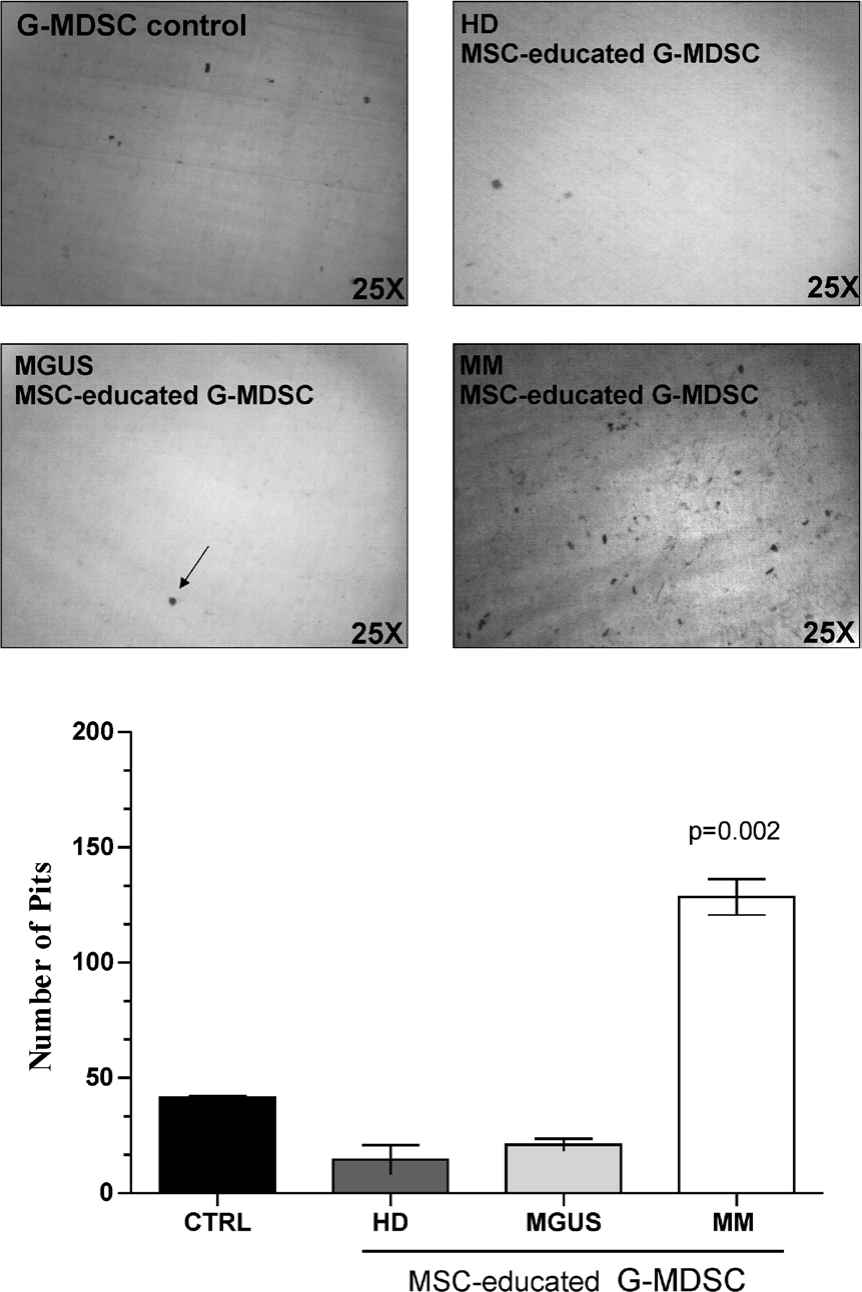
MM-MSCed-G-MDSC acquire bone resorption ability Bone matrix digestion by MM-MSCed-G-MDSC is shown. The number of resorption pits was measured by light microscopy using Image J software. Results are expressed as the number of resorption pits per dentine disks (DDs). A significant digestive activity was observed only in DDs with MM MSCed-G-MDSC compared to G-MDSC control (*p* = 0.002). Values are shown as mean ± SD of three independent experiments in duplicate.

Since MM patients have an enhanced bone resorption and increased inflammatory signals into the microenvironment, we next investigated if MM-MSC-educated G-MDSC may be involved in the digestive process of bone. Therefore, G-MDSC control, HD-, MGUS- and MM-MSCed-G-MDSC were plated onto dentine disks (DDs) for 3 days. A significant digestive activity was observed only in DDs with MM-MSCed-G-MDSC (*p* = 0.002) (Figure 5).

## DISCUSSION

A well-recognized feature of MM is the intimate relationship between PC and BM microenvironment [4]. In this context, it is emerging the role of Myeloid-derived suppressor cells (MDSC) that are able to suppress immune responses, thus inducing an immunosuppressive milieu and contributing to create a permissive microenvironment that fosters evolution of disease [4, 11–17].

There are evidences that MDSC accumulation in PB and BM of MM patients correlates with disease progression and/or drug resistance [5], but little is known about the mechanisms of MDSC expansion, differentiation, and activation in MM. In this paper, we first confirmed that MDSC are increased in MM patients and are able to suppress T cells proliferation. Being MSC a key stromal cell population of the BM milieu that are able to transform the microenvironment into an immunosuppressive one in order to sustain PC proliferation, we have hypothesized the possibility that MSC could contribute to generate MDSC.

In fact, accumulating evidences indicate that tumor-associated microenvironment produces several factors involved in myelopoiesis and impairment of myeloid differentiation. A large amount of molecules released by tumor cells or tumor-surrounding cells, including IL-1β, IL-4, IL-6, IL-10, IFN-γ and TGF-β, are reported to re-program immature myeloid cells to become immunosuppressive [5, 36, 40]. Therefore, we investigated the direct role of MM-MSC in G-MDSC induction. Despite HD-, MGUS- and MM-MSC generate similar amount of G-MDSC, the ability to suppress T-lymphocytes proliferation was found only for G-MDSC that were generated after a co-culture with MSC derived from patients with MM and not from MGUS or healthy subjects. This finding is in line with observations from Sánchez et colleagues who showed that in a murine model oncogenesis drives MSC to increase the production of PGE2 favoring inhibition of lymphocytes proliferation and differentiation of myeloid precursors to highly suppressive cells [41]. However, one of the main question regarding MSC from MM patients stays still whether they are or not different from healthy MSC. Our work contribute to elucidate the different role of MM versus MGUS and healthy MSC, confirming the abnormal immune modulatory ability of MM-MSC. Since this difference in G-MDSC activation has been found for isolated MM MSC after *in vitro* expansion, these stromal cells have a constitutive functional alteration in immune regulation.

When compared with their normal counterparts, MM-MSC differ in cytokine production, show a decreased proliferative ability with a premature senescence profile [42] and reduced capacity to inhibit T cell proliferation [43]. It is still an open question whether MSC have genomic alterations which may contribute to the pathogenesis of MM. MM-MSC exhibit a distinctive gene expression profile compared to HD-MSC [44], suggesting that these differences could be attributed to the presence of genomic alterations in MM counterpart [45, 46]. Some reports have favored the possibility of a common haematopoietic and mesenchymal progenitor [47, 48]. Garayoa et al. tried to find whether cytogenetic abnormalities present in PC were shared by matching MSC from the same patient [27]. All analyzed MM-MSC were cytogenetically normal for the tested genomic alterations, thus excluding a common progenitor for MM-PC and MSCs. With the present work, we suggest that MM-MSC can be functionally different from MSC from healthy subjects and MGUS, favoring immunosuppressive abilities of surrounding myeloid cells.

Exploring the immune-modulatory factors expressed by MM-MSC that are able to generate G-MDSC, we found a statistically significant up-regulation of PTGS2, TGFβ, NOS2, IL10 and IL6 expression, suggesting that multiple mechanisms are involved in generation and activation of G-MDSC. Since gene expression changes were not found at t0, the expression of the immune modulatory factors is influenced by interaction with PBMC *in vitro*, confirming that MM-MSC have constitutive immunological functional alterations.

Comparing MGUS- and MM-MSCed-G-MDSC to HD-MSCed-G-MDSC, both myeloid populations overexpressed IL-6, IL-1-β, PTGS2 and IL-10. However, only MM-MSCed-G-MDSC up-regulated TNFα and ARG1, providing thus evidence that MM-MSC transform myeloid cells in immunosuppressive ones. In fact, up-regulation of ARG1 is one of the main mechanisms of MDSC-induced immune-suppression [49], while TNFα has been shown to arrest differentiation of immature myeloid cells and increase MDSC suppressive activity [50].

Yan et colleagues have also reported that IL-6 cooperates with G-CSF to induce tumor function of murine neutrophils in BM by modulating signaling pathways that favor tumor angiogenesis through up-regulation of PROK2 [51]. Interestingly, we found the up-regulation of PROK2 by MM-MSCed-G-MDSC that may be linked to over-expression of IL6 by MM-MSC during co-culture with PBMC. PROK2 is a key regulator of inflammation-dependent tumorigenesis promoting chemotaxis, angiogenesis [38, 52] and drug-resistance into the tumor microenvironment [53]. Since MGUS-MSCed-G-MDSC do not express PROK2, it is possible that MDSC present in MM patients may contribute to the “angiogenic switch” that characterizes the transition from MGUS to MM.

The osteoblasts derived from MM-MSC exhibit a diminished matrix mineralization potential when compared with MSC from healthy donors [44]. Furthermore, the clinical observation that bone lesions persist in patients who respond to therapy supports the idea of a permanent defect in the MM-MSC [54]. Previous studies have shown that in response to PC, MM associated-mesenchyme release pro-osteoclastogenic cytokines that increase osteoclasts (OC) recruitment and OC-mediated bone loss [55, 56]. STRO-1 is a well characterized MSC antigen expressed by a population of immature and multipotent MSC [57]. Noll et al. demonstrated increased amount of STRO-1 MSC in MM patients that correlated with more severe disease and that expressed higher levels of PC- and OC-activating factors, including RANKL and IL6 [55]. Recently, it has been reported that neutrophils can acquire monocytic characteristics in response to inflammatory signals [58]. For the first time, we demonstrated that, unlike MGUS-, MM-MSCed-G-MDSC were able to digest bone matrix, suggesting that MM-MSC may indirectly contribute to bone resorption by G-MDSC activation.

In conclusion, these findings strongly support an evolving concept regarding the contribution of MM-MSC in tumor development and progression, indicating that cancer progression may rely on MSC both directly and through G-MDSC-mediated immunosuppression into the MM microenvironment, leading to the cancer immune surveillance evasion. The acquisition by MM-MSC of the ability to induce G-MDSC with an immunosuppressive behavior and digestive ability might represent an evolutionary advantage acquired during the multistep development of cancer.

**Figure 6:**
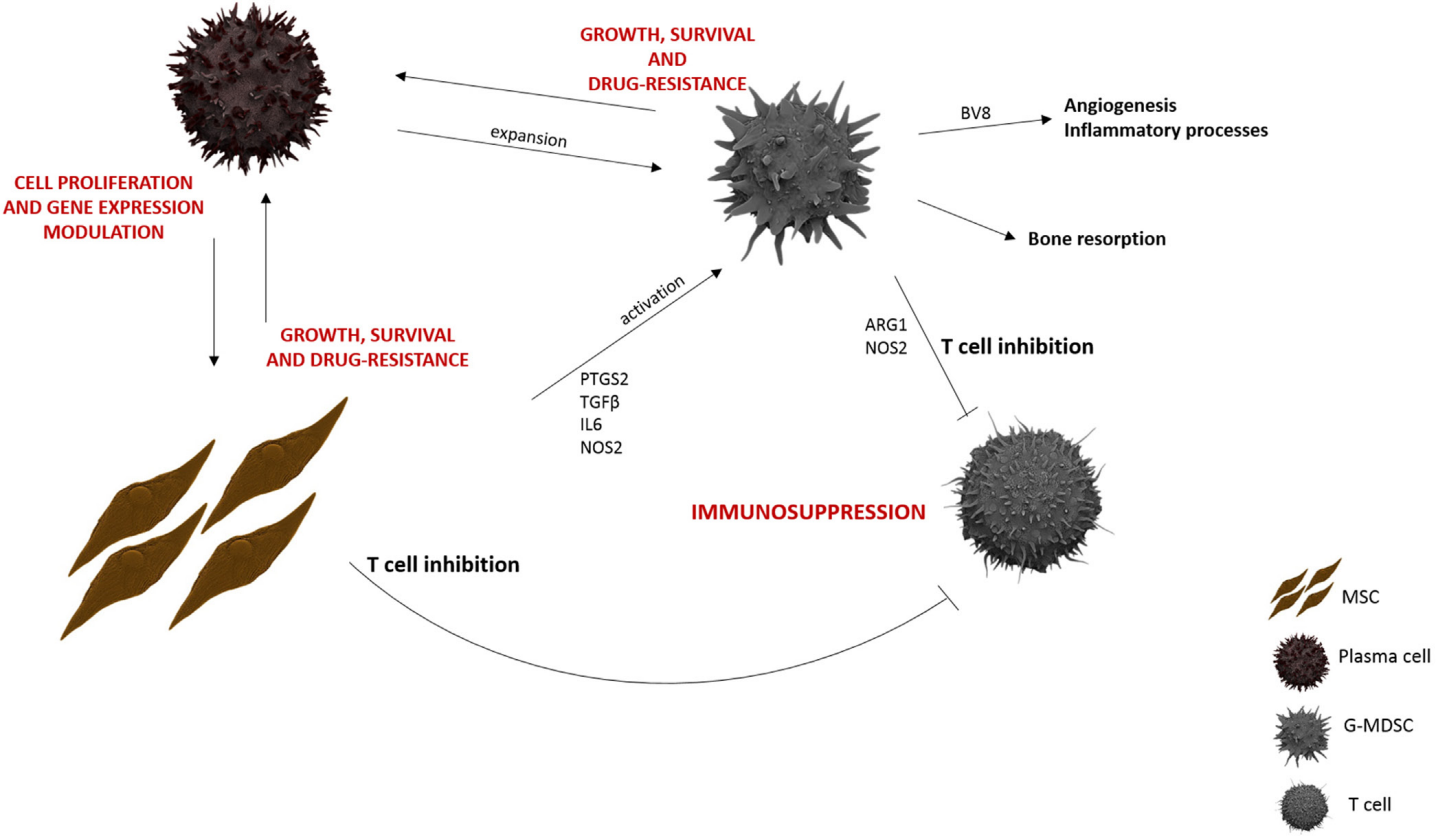
MM BM microenvironment MM-MSC, both directly and through G-MDSC activation, support MM growth, survival and drug-resistance within an immunosuppressive BM microenvironment.

Taken together, our data give further evidence of the key role played by MSC in MM BM microenvironment, making it an immune-tolerant milieu through G-MDSC induction. Therefore, the interaction of MM cells with MSC creates an important loop: PC stimulate the proliferation of MSC [55] and influence their gene expression profile [59], but at the same time MSC support MM cell growth and survival by releasing several cytokines and growth factors, and by promoting MDSC activation (Figure 6). Elucidation of the complex interactions between MM cells and MSC into MM microenvironment is necessary in the development of effective therapies to improve treatment of MM.

## MATERIALS AND METHODS

### Patients and sample collection

After written informed consent approved by the local ethical committee (Azienda ospedaliero Universitaria Policlinico-Vittorio Emanuele, # 34/2013/VE), samples were collected from MGUS (*n* = 30), newly diagnosed (*n* = 30) and relapsedMM (*n* = 15) patients and age-matched HD (*n* = 30) at Division of Hematology, AOU Policlinico–OVE, University of Catania. Clinical data of MM and MGUS patients included in this study are shown in Table 1. Patients were free of immune-mediated diseases and acute or chronic viral infections to exclude any interference on immune-regulatory mechanisms. All MGUS patients had a stable condition with at least 2 years of follow up.

**Table 1:** Baseline clinical characteristics of patients included in the study

	MGUS (*n = 30*)	Newly-diagnosed MM (*n = 30*)	Relapsed MM *(N = 15)*
**Median age (range)**	67 (49–70)	64 (40–81)	67 (38–75)
**Males/Females**	21/9	17/13	8/7
**Istotype, *n***			
*IgG*	0	16	6
*IgA*	6	7	5
*Light-chain only*	0	7	4
**Cytogenetics, *n***			
*Normal*	15	12	1
*del 13*	2	5	4
*del 17*	0	6	4
*t (4;14)*	3	3	4
*not performed/failed*	10	4	2
**Haemoglobin, g/dl (range)**	12.8 (12–14.5)	10.6 (6.5–13.8)	9.8 (6.6–12.8)
**Platelets 1000/uL (range)**	219 (180–315)	221 (90–384)	123 (43–225)
**Bone marrow plasmocytosis > 50%, *n* (%)**	0	12	12
**C-reactive protein median, mg/l (range)**	0.1 (0.01–4)	4.4 (0.01–8.5)	5.3 (0.05–9.6)
**LDH median, U/l (range)**	195 (132–213)	209 (109–708)	240 (125–368)
**ESR median, mm/h**	17 (0–26)	72 (6–134)	84 (10–138)
**STAGE ISS, n**			
*1*	N.A.	7	0
*2*	N.A.	15	10
*3*	N.A.	8	5

WBC: white blood cells count; ALC: absolute lymphocyte count; ESR: Erythrocyte Sedimentation Rate; ISS: International Staging System.

### G-MDSC evaluation

Whole blood collected in EDTA vials (50 μL) was stained with monoclonal antibodies (moAbs, 10 μL for each) and respective isotypic controls [34]. The moAbs (Beckman coulter) included: CD11b FITC (clone bear-1), CD15 PE (clone 80H5), CD14 PC5, (clone RMO52), HLA-DR-ECD (Clone Immu-357). Using sequential gating strategy, G-MDSC were identified as CD11b^+^CD15^+^CD14^−^HLADR^−^. The acquisition and analysis was performed with a Beckman Coulter FC-500 flow cytometer (10,000 cells were analyzed).

To evaluate the suppressive ability, G-MDSC from MM patients and HD were isolated using anti-CD66 magnetic microbeads (Miltenyi Biotec) and then co-cultured for three days with autologous Carboxyfluorescein succinimidyl ester (CFSE)-labeled T lymphocytes at ratio 1:4 [35]. For cell labeling, 5 × 10^5^ lymphocytes were incubated at 37°C for 20 min in 1 ml PBS containing 1 μM CFSE (BD Pharmingen). T cells were stimulated with 5 mg/mL phytohemagglutinin (PHA) and incubated for 72 hours prior to flow cytometry. Controls included a positive T cell proliferation control (T cells plus PHA) and a negative one (T cells only). After three days, T cell proliferation was measured by CFSE dilution and analyzed using flow cytometry.

### MSC harvest and culture

BM mononuclear cells from HD (*n* = 6), MGUS (*n* = 5) and MM (*n* = 7, 4 of which at diagnosis and 3 relapsed) subjects were obtained after density gradient centrifugation on Ficoll and cultured in low-glucose Dulbecco’s modified Eagle’s medium supplemented with 10% heat-inactivated FBS, 100 U/ml penicillin, 100 mg/ml streptomycin and 1% L-glutamine. After 3 days in culture, non-adherent cells were removed, whereas MSCs were selected by their adherence to the plastic-ware. The cultures were maintained at 37°C and 5% CO_2_. MSCs were expanded until the third or fourth passage and then trypsinized to be used for experiments.

Selected MSC from both patients and HD at the third passage were also tested for MSC specific surface antigen expression (Supplementary Figure 3). Therefore, cells were labeled using combinations of monoclonal antibodies: anti-CD34-ECD (clone 581), anti-CD90-FITC (clone F15.42.1.5), anti-CD105-PE (clone 1G2) and anti-CD45-PC5 (clone J.33). The appropriate isotopic control was also included. Labeled MSC were acquired using a Beckman Coulter FC-500 flow cytometer.

### MDSC induction

Human peripheral blood mononucleated cells (PBMC) were isolated from healthy volunteer donors after density gradient centrifugation on Ficoll. PBMC were cultured alone or co-cultured with MSC derived from healthy, MGUS or MM subjects as described above (1:100 ratio). MSC were seeded to achieve confluence by 7 days. After one week, PBMC were collected and G-MDSCs were isolated using anti-CD66b magnetic microbeads. The phenotype of G-MDSC was confirmed by cytofluorimetric analysis. Their immunosuppressive capacity was analyzed by evaluating T cell anergy when co-cultured with autologous CFSE-labeled T cells stimulated by PHA. Controls included a positive T cell proliferation control (T cells plus PHA) and a negative one (T cells only). After three days T cell proliferation was analyzed by flow cytometry.

### Real-time RT-PCR for gene expression of MSC and MDSC

For gene expression studies, MSC were trypsinized from culture flasks both at Time 0 (cells at confluence incubated with standard medium only) and after 48 hours from start of co-culture experiments. In addition, MSC co-cultured with PBMC were purified using anti-CD271 magnetic microbeads. G-MDSC control (isolated from PBMC cultured in medium alone), MGUS- and MM-MSC-educated G-MDSC were collected from co-cultures and isolated by magnetic microbeads. After RNA extraction and reverse transcription, we evaluated expression of the following mRNA: ARG1 (Arginase 1), NOS2 (Nitric Oxide Synthase 2), PTGS2 (Prostaglandin-Endoperoxide Synthase 2), TNFα (Tumor necrosis factor alpha), TGFβ (Transforming growth factor beta), IL6 (Interleukin 6), IL10 (Interleukin 10), IL1β (Interleukin 1beta), and PROK2 (Prokineticin 2/BV8). Their expression was assessed by TaqMan Gene Expression (Life Technologies) and quantified using a fluorescence-based real-time detection method by 7900HT Fast Start (Life Technologies). For each sample, the relative expression level of each studied mRNA was normalized using GAPDH as invariant controls.

### Resorption assay

MSC-educated G-MDSC (2 × 10^4^/cm^2^) were plated onto dentine discs (Osteosite Dentine Discs (DDs), Immunodiagnostic Systems Inc., Fountain Hills, AR) in 96-well plates for the digestion test. After 96 h, the cells were removed with 5% sodium hypochlorite for 10 min. Discs were rinsed with water and stained with 1% (w/v) toluidine blue in 0.5% sodium borate for 30 s and then washed twice with water. The number and the area of resorption pits were then measured by light microscopy using ImageJ software. Results were expressed as the number of resorption pits. Experiments were performed in triplicate.

### Statistical analysis

Statistical analyses were made with Prism Software (Graphpad Software Inc., La Jolla, CA, USA). Data were expressed as mean or SD. Statistical analysis was carried out by unpaired *t*-test or ANOVA test. A *p*-value of 0.05 was considered to indicate a statistically significant difference between experimental and control groups.

## SUPPLEMENTARY MATERIALS FIGURES


