# Supplementary material for: Granulocyte-like myeloid derived suppressor cells (G-MDSC) are increased in multiple myeloma and are driven by dysfunctional mesenchymal stem cells (MSC)
Source: Oncotarget. 2016 Mar 7;7(52):85764–75. doi: 10.18632/oncotarget.7969 (PMC5349872; doi:10.18632/oncotarget.7969)
Supplement: Supplementary file 1 [file oncotarget-07-85764-s001.pdf]

Granulocyte-like myeloid derived suppressor cells (G-MDSC) are increased in multiple myeloma and are driven by dysfunctional mesenchymal stem cells (MSC)

Supplementary Materials

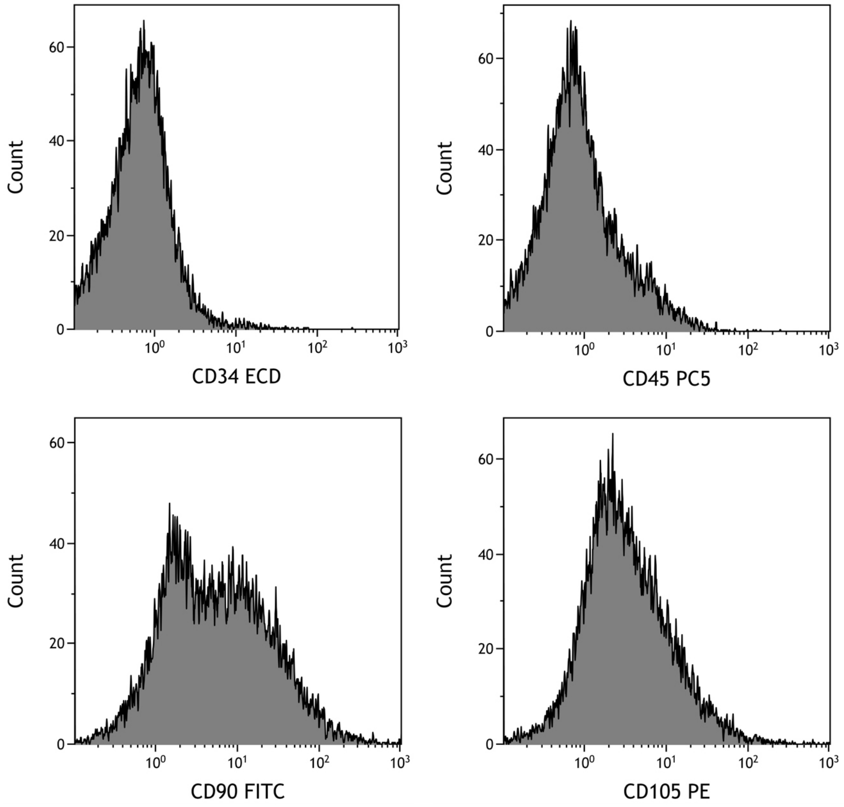

Supplementary Figure S1: Evaluation of MSC specific surface antigen expression. Representative data from one MM-MSC sample. Flow cytometry analysis shows that MSC are positive for CD90 and CD105 negative for CD34 and CD45.

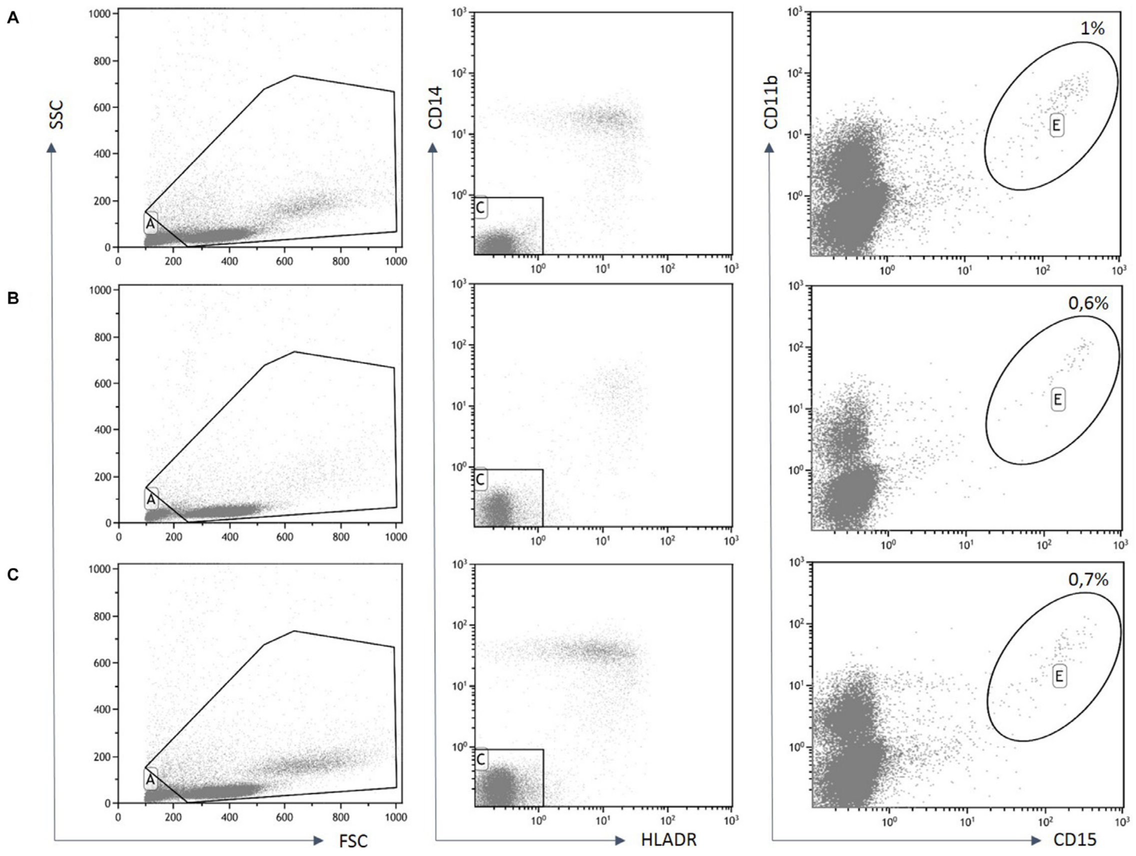

Supplementary Figure S2: HD-, MGUS- and MM-MSC generate similar amount of G-MDSC. The figure shows a representative data from one experiment. (**A**) HD-MSC; (**B**) MGUS-MSC; (**C**) MM-MSC. Flow cytometry analysis was performed with gates set on CD11b+ CD15+CD14-HLADR- cell population.

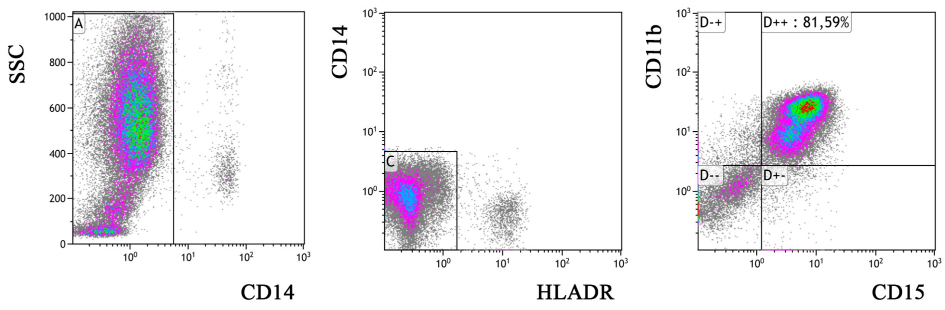

Supplementary Figure S3: Purity of educated G-MDSC after magnetic cell separation. After separation, the cells were incubated with fluorescently labeled anti-CD11b, anti-CD15, anti-CD14 and anti-HLADR antibodies, and the purity of the cells was analyzed by flow cytometry. The figure reports the representative flow cytometry dot plots showing the purity of educated G-MDSCs.
